# Case report: Allogeneic feline umbilical cord-derived mesenchymal stem cell transplantation for feline oral squamous cell carcinoma

**DOI:** 10.3389/fvets.2024.1443110

**Published:** 2024-07-23

**Authors:** Mi-Kyung Park, Kun-Ho Song

**Affiliations:** ^1^Department of Veterinary Internal Medicine, College of Veterinary Medicine, Chungnam National University, Daejeon, Republic of Korea; ^2^CM Animal Hospital, Jincheon, Republic of Korea

**Keywords:** feline, umbilical cord, mesenchymal stem cell, oral squamous cell carcinoma, anti-inflammatory effect

## Abstract

A 5-year-old neutered female Korean domestic shorthair cat diagnosed with oral squamous cell carcinoma (SCC) presented to the hospital with severe oral purulent discharge, anorexia, and lethargy. Owing to extensive lesions, surgical excision and radiation therapy were not feasible. Instead, prior to metronomic therapy with toceranib, the patient received an intravenous injection of feline umbilical cord-derived mesenchymal stem cells (fUC-MSCs) (1 × 10^6^ cells/10 mL of saline) to reduce inflammation. No acute side effects (such as fever, increased respiratory rate, diarrhea, and vomiting) were observed following stem cell therapy. For 6 days, purulent discharge, bleeding, swelling, a bad odor, and crust exfoliation in the tumor area on the face were dramatically reduced. However, the patient exhibited difficulty in voluntarily receiving foods, and weight loss persisted. Starting from the 7th day, purulent discharge, bleeding, and odor at the SCC area worsened again. Toceranib, low-dose NSAIDs (meloxicam, every other day), antibiotics (cefazoline), and gabapentin were administered; however, they were not effective in reducing the pus, bleeding, foul odor, and crust exfoliation at the SCC area. Symptoms of pain, weakness, and weight loss progressed, leading to the choice of euthanasia with the owner’s consent approximately 1 month later. This case report reveals that allogeneic fUC-MSCs have a slight short-term effect on purulent discharge, bleeding, odor, and crust exfoliation and may be additional therapy for feline oral SCC.

## 1 Introduction

Feline oral squamous cell carcinoma (FOSCC) is the most common malignant oral tumor in cats, accounting for more than 60–70% of all feline oral tumors (1, 2). Although the etiology of feline SCC is not fully understood, it is thought to be caused by multiple variables, such as environmental factors (3, 4). Without appropriate treatment, the average survival time following diagnosis of FOSCC is less than 2 months, indicating a significantly poor prognosis (5). Treatment options include surgical resection, radiation therapy, and chemotherapy; however, the efficacy and side effects of radiation therapy and chemotherapy depend on the stage of SCC (6–9). Recently, toceranib combined with low-dose NSAID have been reported to improve the survival time of cats with OSCC (10). Accordingly, patients with FOSCC have a very poor quality of life and poor prognosis, severe pain, eating disorders, inflammation, and bleeding.

Recently, mesenchymal stem cells (MSCs) have received significant attention for the treatment of various diseases owing to their anti-inflammatory, immunomodulatory, antioxidant, tissue regenerative, and anticancer effects in human medicine (11–14). MSCs can be easily obtained from various tissues, such as bone marrow, adipose tissue, and fetal tissues (15). In particular, the safety and efficacy of human umbilical cord stem cells have been evaluated extensively in preclinical and clinical studies (16). They exert beneficial effects in various diseases, such as stroke, Parkinson’s disease, Alzheimer’s disease, and diabetes (17). Ongoing research on human umbilical cord stem cells is focused on their anticancer effects on various cancer cells, such as breast cancer, prostate cancer, lung cancer, Burkitt’s lymphoma, and liver cancer (18–20). Recently, an *in vitro* study revealed that human umbilical cord MSC-derived exosomes have positive effects in oral SCC (21). Additionally, recent report demonstrated that human menstrual stem cell exosomes inhibit angiogenesis and tumor growth of oral squamous cell carcinoma (22). In the veterinary field, active research is focused on stem cells and their applications across various diseases. Specifically, in felines, the efficacy of adipose-derived and bone marrow-derived stem cells has been demonstrated in various diseases, such as gingivostomatitis, asthma, and chronic kidney disease (23–25). Additionally, recent studies have highlighted the antioxidant properties of feline umbilical cord stem cells via the NF-kB pathway *in vitro* (26). However, there is lack of clinical research regarding the safety and efficacy of feline umbilical cord stem cells in various diseases (27).

In this case study, we administered allogeneic feline umbilical cord MSCs (fUC-MSCs) to a patient diagnosed with FOSCC experiencing severe inflammation, bleeding, and pain before toceranib chemotherapy.

## 2 Case description

**Figure 1:**
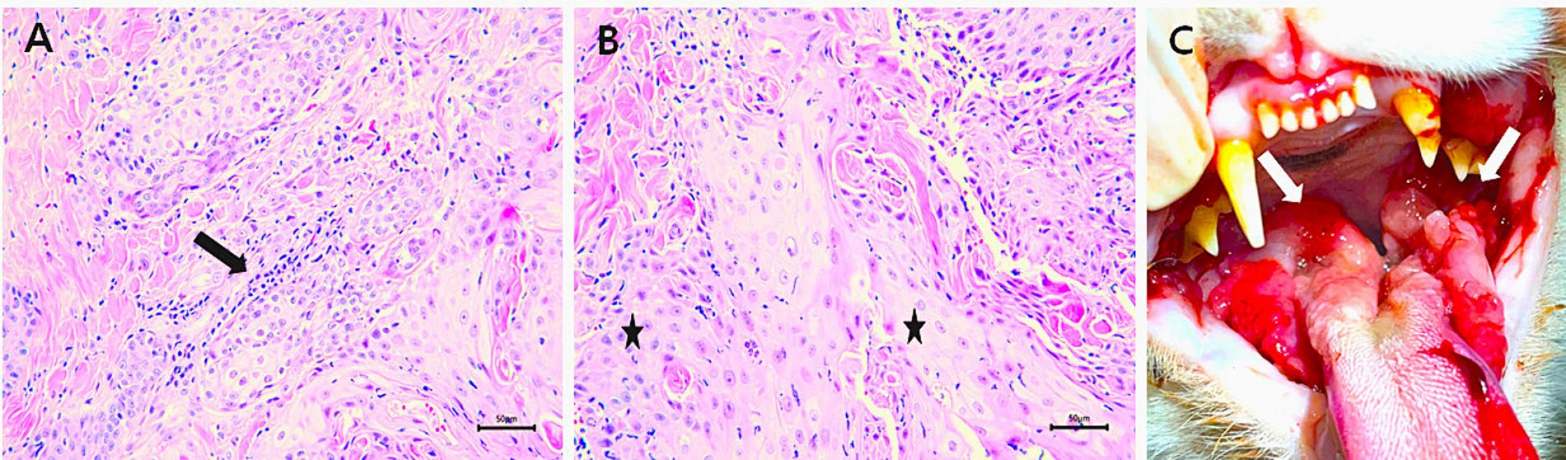
Examinations of the patient. **(A)** Mild inflammation (arrow) due to FCGS was observed within the lesion; ×400 magnification; scale bar, 50 μm. **(B)** Poorly differentiated feline oral squamous cell carcinoma cells (FOSCC) (*); ×400 magnification; scale bar, 50 μm. **(C)** Intraoral photograph of patient with extensive oral ulceration and severe inflammation lesions in the tongue, and oral mucosa on both sides due to FCGS (arrows).

A 5-year-old neutered female Korean domestic shorthair cat weighing 3 kg was presented to our animal hospital after being diagnosed with FOSCC based on a histological examination at another animal hospital (Figures 1A,B). The patient was diagnosed with feline chronic gingivostomatitis (FCGS), characterized by excessive salivation, severe inflammation of oral cavity at our animal hospital 1 year before. Upon visual inspection, extensive oral ulceration and inflammation lesions were observed in the tongue, and oral mucosa on both sides (right and left mandible) (Figure 1C). Two months ago, the patient’s right maxillary fourth premolar tooth was extracted due to severe inflammation and increased mobility at our hospital. About a month after the tooth extraction, the patient presented with rapidly growing mass on the face and subsequently revisited our hospital. During palpation, a large and solid mass extending from the site of the tooth extraction to the face was identified. The patient exhibited severe oral purulent discharge with bleeding, foul odor, crust exfoliation on the right side of the face, frequent head shaking, lethargy, pale mucous membranes, enlargement of mandibular lymph nodes, pallor, and anorexia. The patient had difficulty eating on its own. The patient tested negative for the feline leukemia virus and feline immune deficiency virus (FeLV/FIV) and the body temperature was within the normal range. Additionally, no metastatic diseases were found in the patient’s whole-body radiography and abdominal ultrasonography.

**Figure 2:**
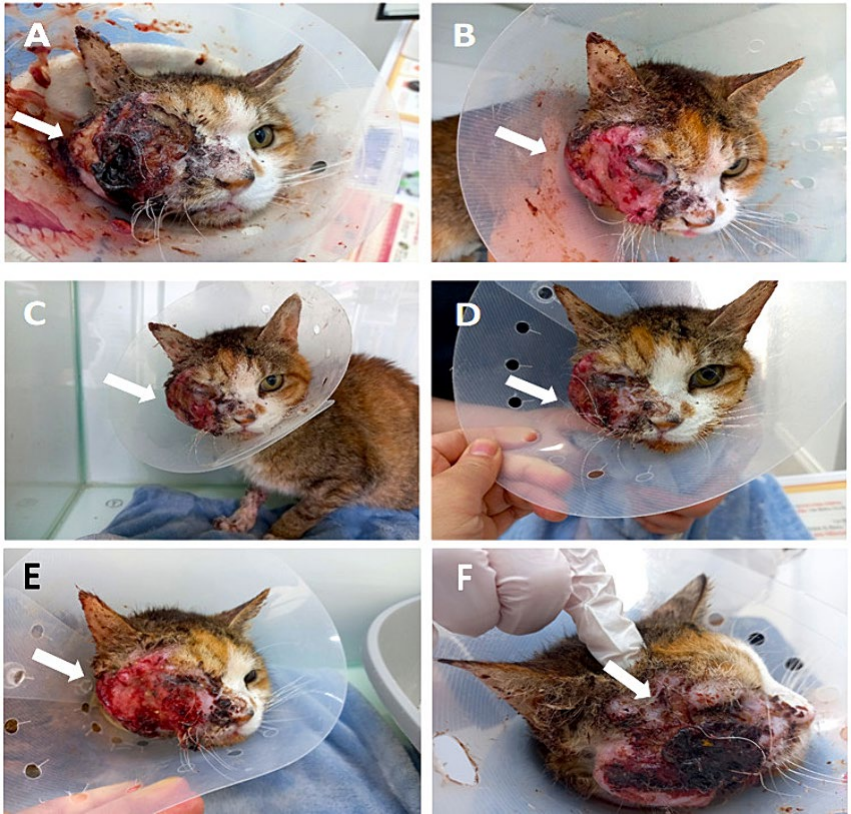
Facial inflammation before and after MSC transplantation, showing a decrease in facial inflammation (FeDESI scores). **(A)** Before the injection, the facial inflammation was severe, and bleeding and crust exfoliation occurred several times a day (arrow) (FeDESI score 30). **(B)** At 12 h after the injection, inflammation and bleeding of the face drastically decreased, and crust exfoliation did not occur (arrow) (FeDESI score 14). **(C)** At 1 day after the injection, the improved state was maintained (arrow) (FeDESI score 14). **(D)** At 6 days after the injection, purulent discharge of the face increased again (arrow) (FeDESI score 16). **(E)** At 10 days after the injection, the erythema and excoriation of the face worsened (arrow) (FeDESI score 20). **(F)** At 28 days after the injection, severe inflammation of the face (arrow) (FeDESI score 30).

Blood tests revealed an elevated leukocyte count (35.3 × 10^9^/L) [reference interval (RI): 5.5–19.5/L], low red blood cell count (4.56 × 10^12^/L) (RI: 4.6–10/L), low hemoglobin (6.8 g/dL) (RI: 9.3–15.3 g/dL), low hematocrit (20.1%) (RI: 28–49%), elevated serum total calcium (14.9 mg/dL) (RI: 8.9–12.6 mg/dL), elevated globulin (5.7 g/dL) (RI: 2.6–5.1 g/dL), elevated amylase (2,500 U/L) (RI: 500–1,400 U/L), elevated lipase (42 U/L) (RI: 0–32 U/L), low sodium ions (134 mmol/L) (RI: 140–160 mmol/L), low chloride ions (101 mmol/L) (RI: 109–128 mmol/L), and an elevated Feline serum amyloid A level (26.6 μg/mL) (RI: 0–5 μg/mL). Additionally, other serum biochemistry levels [blood urea nitrogen (BUN), creatinine, serum phosphorus, alanine amino transferase (ALT), alkaline phosphatase (ALP), albumin (ALB), glucose (GLU), cholesterol, total bilirubin (TBIL)] were normal range. No further blood tests were performed at the owner’s request. Although antibiotic (cefazolin, 20 mg/kg, PO, q12h), anti-inflammatory drugs [prednisolone (0.5 mg/kg, SC, q12h), dexamethasone (0.1 mg/kg, SC, q12h)], NSAID (meloxicam, 0.04 mg/kg, SC, q24h), pain management (gabapentin, 10 mg/kg, PO, q12h), and fluid therapy were administered for 7 days, the patient showed no improvement, and inflammation at the SCC site worsened (Figure 2A). Owing to the extensive span of the SCC tumor mass (about 4 × 4 cm), surgical removal and radiation therapy would be difficult, and treatment with toceranib phosphate and low-dose NSAIDs was recommended. The patient experienced severe pain, repeated epistaxis, and facial inflammation, leading to a loss of appetite and forced feeding. Before toceranib arrived at the hospital, MSC therapy was initiated with the owner’s consent to reduce inflammation at the site of facial SCC urgently.

**Figure 3:**
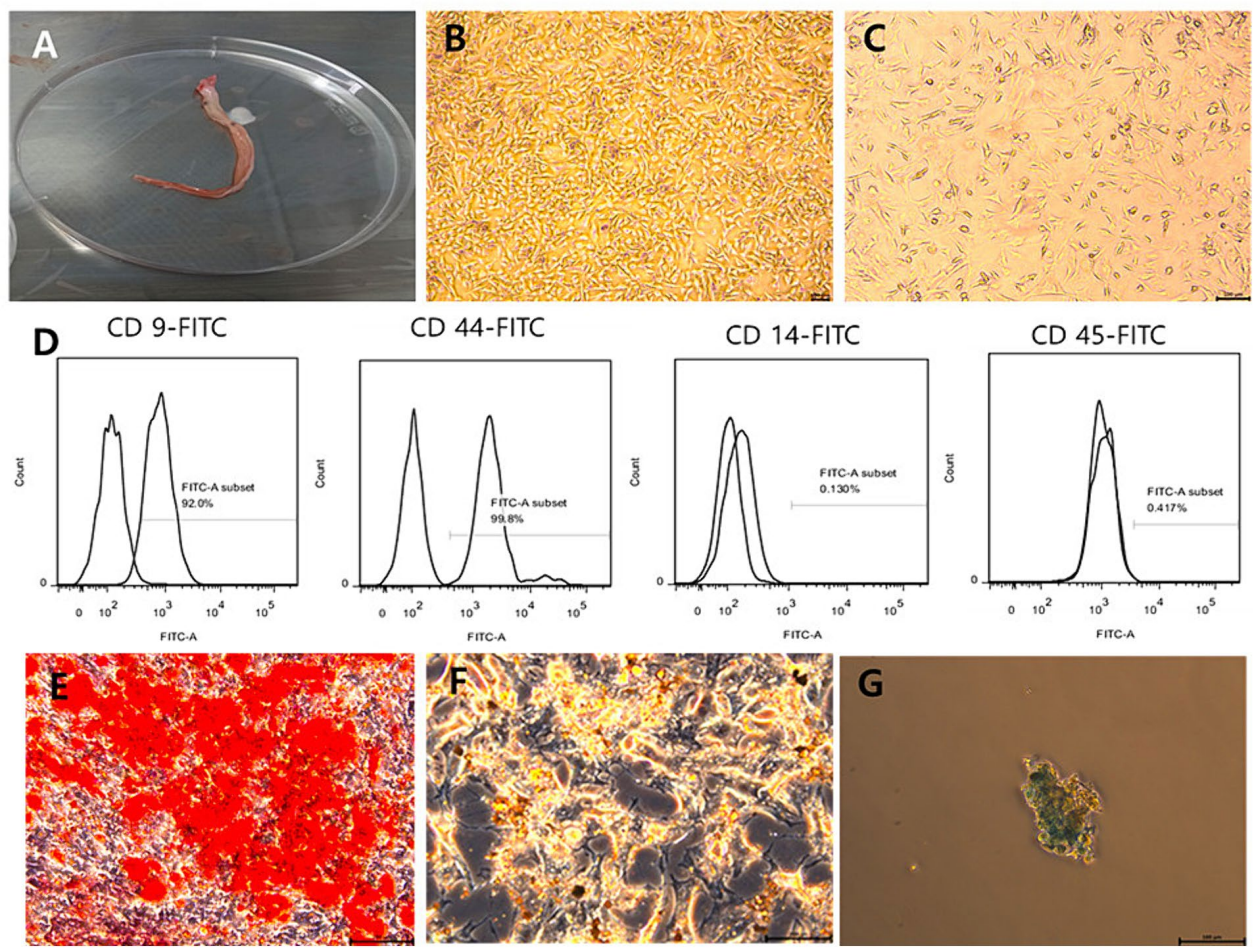
Feline umbilical cord tissue and characterization of fUC-MSCs. **(A)** Feline umbilical cord tissue. **(B)** × 50 magnification; **(C)** × 100 magnification; Passage 3 cells show a spindle-shaped morphology. **(B,C)** Scale bars, 100 μm. **(D)** Immunophenotype of fUC-MSCs determined by flow cytometry. **(E)** Osteogenic differentiation, evaluated using Alizarin Red S staining. **(F)** Adipogenic differentiation, evaluated using Oil Red O staining. **(G)** Chondrogenic differentiation, evaluated using Alcian Blue staining. **(E–G)** Scale bars, 100 μm.

Feline umbilical cords (*n* = 5) were collected during a cesarean section from a healthy female domestic shorthair cat aged 1 year (Figure 3A). The donor cat was patient of our animal hospital. The cat was showed normal blood analysis and imaging finding and the FeLV/FIV test was negative. Immediately after dissection, umbilical cords were aseptically collected, rinsed three times in phosphate-buffered saline (PBS) (Welgene, Gyeongsan-si, Korea), and placed in conical tubes contained PBS and 1% penicillin/streptomycin (Welgene, Gyeongsan-si, Korea). The protocols for this study followed the guidelines of the Animal Care and Use Committee of Chungnam National University (approval number: 202212A-CNU-250).

To obtain the cells, the tissue was triturated and digested with Collagenase type І (1 mg/mL; Sigma-Aldrich, St. Louis, United States), and the mixture was shaken every 15 min for 45 min at 37°C in a humidified atmosphere of 5% CO_2_. After enzyme incubation, the cell suspensions were filtered through a 100 μm cell strainer (Corning, Inc., Corning, NY, United States). After washing the cells three times by centrifugation, the pellet was resuspended in culture medium containing Dulbecco’s modified Eagle’s medium supplemented with 15% Fetal Bovine Serum (Gibco, Waltham, MA, United States), 1% GlutaMax (Gibco), and 1% penicillin/streptomycin. Cells were cultured under sterile conditions in T-75 flasks under a humidified atmosphere of 5% CO_2_ at 37°C for 7 days. The culture medium was changed twice per week and passed until the adherent cells reached 70–80% confluence. We then isolated MSCs from tissues and evaluated the morphology of MSCs at passage 3. The cells generally exhibited a spindle-shaped morphology under a microscope (Figures 3B,C). To confirm that the feline umbilical cord-derived cells were MSCs, their phenotypes were analyzed using flow cytometry. The cells were stained with the following antibodies: Fluorescein isothiocyanate (FITC) − conjugated against CD9 (antibody clone MM2/57, MA5-16860, Invitrogen, United States), FITC−conjugated against CD14 (antibody clone TüK4, MA1–82074, Invitrogen, United States), FITC−conjugated against CD44 (antibody clone IM7, MA1–10229, Invitrogen, United States), FITC−conjugated against CD45 (antibody clone YKIX716.13, 11–5,450 − 42, Invitrogen, United States). The cells analyzed using a BD Canto II flow cytometer (BD Biosciences, USA). The results were evaluated using the FlowJo software (Tree Star, USA). The cells were positive for the MSC markers CD9 and CD44 and negative for CD14 and CD45, which are hematopoietic cell markers (Figure 3D). When cells were cultured in specific differentiation media, MSCs exhibited the ability to differentiate into adipocytes, osteoblasts, and chondroblasts (Figures 3E–G).

Passage 3 fUC-MSCs were slowly administered intravenously 1 × 10^6^ cells/10 mL of saline (0.33 × 10^6^ cells/kg) for 30 min. No acute adverse reactions (such as fever, increased respiratory rate, diarrhea and vomiting) were observed during the 12 h monitoring period after the injection (28). Twelve hours after the injection, bleeding and facial purulent discharge decreased, and crust exfoliation did not occur (Figure 2B). The cat then exhibited increased activity, vocalizations, and responsiveness to humans. In addition, the severe odor of the face decreased drastically. However, the patient remained unable to eat voluntarily and was force fed. On Day 1 after the injection, the slight improvement in activity and reduction in purulent discharge were maintained (Figure 2C). Also, facial bleeding, purulent discharge, swelling, and odor decreased drastically, and there was no crust exfoliation observed.

The effect lasted 6 days. On Day 6, purulent discharge at the facial SCC site began to increase (Figure 2D). On Day 10, the erythema and excoriation of the face worsened (Figure 2E). So, toceranib (3.25 mg/kg, PO, q48h) was administered with low-dose NSAIDs (meloxicam, 0.01 mg/kg, SC, q24h) and gabapentin (10 mg/kg, PO, q12h). However, the facial purulent discharge gradually worsened, and the patient experienced anorexia, severe pain, and rapid weight loss to 1.68 kg over 28 days (Figure 2F). On Days 0, 1, 6, 10, and 28, the skin inflammatory lesions of the patient’s face were assessed by a veterinarian using the Feline Dermatitis Extent and Severity Index (FeDESI). Three types of lesions (erythema, excoriation and alopecia) were graded 0 (normal), 1 (mild), 3 (moderate) or 5 (severe) at 42 different body sites to give a final score to from 0 to 630 (29, 30). The FeDESI scores significantly decreased from 30 (Day 0) to 14 (Day 1). However, the FeDESI scores began to increased slightly from 16 (Day 6) to 20 (Day 10) and to 30 (Day 28). Additionally, On Days 0, 1, 6, 10, and 28, the patient’s pain was evaluated using the Glasgow composite pain scale (range 0–20) (31). The pain scores greatly decreased from 16 (Day 0) to 10 (Day 1) and to 10 (Day 6) but increased from 12 (Day 10) to 18 (Day 28). Considering the patient’s pain and quality of life, we recommended euthanasia to the owner. One month later, the patient was euthanized after consent was obtained from the owner.

## 3 Discussion

Feline oral SCC is a frequent oral cancer (1). Although there are treatments, such as surgery, radiation therapy, and chemotherapy, the prognosis is poor (5). FOSCC is difficult to successfully treat due to its high invasion and recurrence rates (1, 2). Owing to the anatomical structure of the oral cavity, surgery and radiation therapy may be difficult depending on the extent of the tissue, and chemotherapy (such as doxorubicin, cyclophosphamide, carboplatin, and zoledronate) is highly toxic and has side effects, resulting in a poor prognosis (6–9). In addition, SCC causes severe inflammation, bleeding of the oral cavity, and severe pain, making eating difficult, which negatively impacts the quality of life of owner(s) and patient. Therefore, FOSCC requires not only conventional therapy (such as surgery, chemotherapy and radiation therapy) but also additional treatment for inflammation, bleeding, and pain. There is an urgent need to develop various additional therapies for FOSCC.

Currently, research on the treatment of various diseases using MSCs is active in human medicine (32). MSC therapy is recognized for its anti-inflammatory and immunomodulatory effects achieved through cell-to-cell contact and the secretion of various factors by MSCs (33). In particular, human umbilical cord mesenchymal stem cells (hUC-MSCs) exhibit excellent anti-inflammatory, immunomodulatory, and anticancer effects, making them a promising treatment for various inflammatory diseases and cancers (34–38). Moreover, hUC-MSCs have immune tolerance, making them advantageous for injection and banking (36). Unlike bone marrow and adipose tissue, umbilical cord stem cells are obtained from fetal by-products after birth without anesthesia or invasive procedures (such as fat tissue biopsy and bone marrow aspiration), making them an accessible and acquiring large quantities of fresh MSCs (39). Recently, an *in vitro* study revealed that hUC-MSC-derived exosomes reduce inflammation and induce apoptosis in oral SCC (13). However, in the veterinary field, research on fUC-MSC treatment for cancer is still insufficient (26).

This case suggests that fUC-MSCs transplantation temporarily alleviates severe inflammation in FOSCC. As a result, it was observed that bleeding, purulent discharge, swelling, erythema and crust exfoliation in the face were reduced dramatically and pain also decreased over 6 days. Purulent discharge started again on the 7th day after the fUC-MSC injection and erythema, excoriation and pain increased. The patient had severe anemia and dehydration due to eating disorders, which would have reduced the response to various treatments. In order to alleviate eating disorders and dehydration caused by the patient’s pain, gabapentin, NSAIDs and fluid therapy were administered, but there was no improvement in symptoms. Although parathyroid hormone and calcitriol were not measured, a paraneoplastic syndrome cause by FOSCC, was found in the patient (40). We treated prednisolone, dexamethasone and fluid therapy for hypercalcemia, but did not improve (41).

While observing the results of this case, we raised several questions: (1) Did fUC-MSCs reduce inflammation in FOSCC while simultaneously contributing to angiogenesis and tissue regeneration? (2) fUC-MSCs showed an anti-inflammatory effect in a small number of 0.33 × 10^6^ cells/kg, compared to the typical range of 1–4 × 10^6^ cells/kg used in feline stem cell therapy studies (22); in clinical practice, would other feline mesenchymal stem cells (such as bone marrow, adipose, amniotic membrane, amniotic fluid) also show anti-inflammatory effects in a few transplanted cells in FOSCC? (3) Although prednisolone, dexamethasone, or NSAIDs did not reduce inflammation in FOSCC, fUC-MSCs exhibited anti-inflammatory effects; what components of fUC-MSCs differentiate them from other drugs?

This case report had several limitations. First, as this was one case, it is difficult to draw conclusions regarding fUC-MSCs therapy. Second, although approximately 30 days of post-treatment follow-up showed no side effects, the case did not survive to evaluate the long-term side effects. Third, since treatments (toceranib and low-dose NSAIDs) were administered after MSCs transplantation, we cannot confirm that MSCs transplantation had a definite effect on improvement of patient’s clinical signs.

Taken together, this case suggests that fUC-MSCs exert short-term positive effects and pain relief in FOSCC. Further studies are needed to explore the therapeutic value of fUC-MSCs, including studies of its long-term safety, the relationship between the number of transplanted cells and efficacy, and anti-inflammatory mechanisms of action. Additionally, further studies are required to evaluate the effects of fUC-MSCs therapy combined with conventional therapies, such as chemotherapy and radiation therapy, in FOSCC.

In conclusion, this case report suggests that fUC-MSCs warrants further evaluation as a beneficial additional anti-inflammatory therapy in FOSCC.

## Data availability statement

The raw data supporting the conclusions of this article will be made available by the authors, without undue reservation.

## Ethics statement

The animal studies were approved by the Animal Care and Use Committee of Chungnam National University (approval number: 202212A-CNU-250). The studies were conducted in accordance with the local legislation and institutional requirements. Written informed consent was obtained from the owners for the participation of their animals in this study.

## Author contributions

M-KP: Conceptualization, Data curation, Investigation, Methodology, Writing – original draft. K-HS: Conceptualization, Supervision, Validation, Writing – original draft, Writing – review & editing.
